# Doença de Kawasaki: Preditores de Resistência à Imunoglobulina Intravenosa e Complicações Cardíacas: Novas Perspectivas?

**DOI:** 10.36660/abc.20201353

**Published:** 2021-03-03

**Affiliations:** 1 Universidade Federal da Bahia Faculdade de Medicina da Bahia SalvadorBA Brasil Universidade Federal da Bahia - Faculdade de Medicina da Bahia, Salvador, BA – Brasil

**Keywords:** Síndrome do Linfonodo Mucocutâneo/tendências Doença de Kawasaki, Artéria Coronária/anormalidade, Vasculite, Crianças, Imunoglobulinas, Intravenosas, Resistência a Drogas

A doença de Kawasaki (DK) é uma doença inflamatória aguda de origem desconhecida, associada à vasculite que afeta vasos de médio calibre. As anormalidades da artéria coronária (AAC) – aneurismas ou dilatações – representam as principais complicações da DK, sendo, na atualidade, a causa mais comum de doença cardíaca adquirida em crianças em países desenvolvidos. Durante a fase aguda, o tratamento padrão inicial recomendado é o uso de imunoglobulina intravenosa (IGIV) e ácido acetilsalicílico, no intuito de reduzir o risco de danos às artérias coronárias.^1^ Contudo, cerca de 10% a 20% das crianças com DK não respondem ao tratamento padrão inicial, apresentando febre recorrente dentro de 36 a 48 horas após infusão da IGIV. Estudos demonstram que a probabilidade de pacientes com DK resistentes à IGIV terem lesões nas artérias coronárias é nove vezes maior do que nos casos sensíveis à IGIV. Sugere-se existir uma janela crítica para bloquear o processo inflamatório e prevenir AAC a longo prazo.^2^

O estudo de Faim et al.,3 teve como objetivo identificar fatores preditores de resistência à IGIV, calcular a eficácia dos modelos preditores japoneses e caracterizar as complicações cardíacas dos pacientes acompanhados em uma única instituição.^3^

Dos 48 pacientes com DK incluídos no estudo entre 2006 a 2018, 17% tiveram diagnóstico de DK atípica. A totalidade apresentava febre no dia da admissão, com média de 5 dias, e todos fizeram tratamento na fase aguda com IGIV e ácido acetilsalicílico, com média de 6,5 dias. Resistência à IGIV foi descrita em 9 casos (21%), dos quais um de DK atípica. São achados compatíveis com dados de literatura.^4^

No intuito de identificar precocemente os casos resistentes ao tratamento com IGIV e, dessa forma, possibilitar a introdução precoce de outras estratégias de tratamento, estudos têm sido realizados tentando estabelecer critérios clínicos e laboratoriais preditores de resistência à IGIV, como idade, dosagem de albumina, transaminases, hemoglobina, proteína C-reativa (PC-R), velocidade de hemossedimentação (VHS), plaquetas, bilirrubinas, sódio, entre outras.^4^ No presente estudo, os autores construíram curvas ROC para encontrar fatores preditores de resistência e elaboraram modelo preditor utilizando regressão logística multivariada. Analisando as variáveis preditoras de resistência, a PC-R apresentou AUC ROC = 0,789; ponto de corte = 15,1 mg/dL; sensibilidade (S) = 77,8%; e especificidade (E) = 78,9%. A VHS apresentou AUC ROC = 0,781; ponto de corte = 90,5 mm/h; S = 66,7%; e E = 85,7%. O modelo preditor com as duas variáveis apresentou valor p = 0,042 e AUC ROC = 0,790.

Os dados da literatura são controversos, quanto ao uso da VHS como variável preditora de resistência à IGIV. Em metanálise, Baeck et al.,^5^ tendo como objetivo identificar fatores laboratoriais preditivos de resistência à IGIV, incluíram 12 estudos publicados entre 2006 e 2014, analisando 2.745 pacientes. Destes, sete estudos calcularam o tamanho do efeito da VHS como fator preditivo de DK resistente à IGIV. A heterogeneidade entre esses estudos foi baixa (Q (6) = 11,001, P >0,001, I2 = 45,459) e a metanálise demonstrou que o tamanho do efeito foi pequeno para VHS (efeitos aleatórios, 0,150).^5^

Em metanálise realizada por Xuan Li et al., avaliando 28 estudos envolvendo 26.260 pacientes, cerca de 4.442 pacientes tinham diagnóstico de DK resistente à IGIV e 21.818 pacientes DK sensíveis à IGIV. A metanálise mostrou que a VHS no grupo resistente à IGIV foi significativamente maior do que no grupo sensível à IGIV. Contudo, a forte associação entre VHS e DK foi demonstrada somente em dois estudos em população chinesa, não sendo evidenciados os mesmos resultados em coreanos, japoneses e não asiáticos. Os demais estudos mostraram fraca associação entre valores de VHS e resistência à IGIV.^6^

Na diretriz sobre doença de Kawasaki publicada em 2017 pela American Heart Association,^1^ destaca-se que o valor do VHS aumenta com IGIV, não devendo ser utilizada para avaliar resposta terapêutica, e que a VHS persistentemente elevada isoladamente não deve ser interpretada como sinal de resistência à terapia com IGIV (Classe III, NE: C).

Desse modo, devemos analisar com cautela o uso dos valores da VHS como preditor de risco independente para resistência à IGIV na população não asiática.

Quanto aos três modelos de escores de risco desenvolvidos – Kobaysahi et al.,^7^ Sano et al.,^8^ e Egami et al.,^9^ validados para a população japonesa^7^^–^^9^ –, eles não apresentam desempenho adequado em populações ocidentais, etnicamente mistas e chinesas.^10^^–^^14^ Situação semelhante foi observada por Faim et al.,^3^ em que os respectivos modelos validados no Japão também não apresentaram desempenho adequado.^3^

Vale destacar no estudo de Faim et al.,^3^ que cerca de 25% dos pacientes apresentaram envolvimento coronário, 40% destes com formação de aneurismas, além de outras complicações cardiovasculares. AAC e outras complicações cardiovasculares na fase aguda, como miocardite, choque cardiogênico e derrame pericárdico, são descritas na literatura.^1^^,^^2^^,^^4^

Independentemente do tamanho da amostra analisada e de ser estudo retrospectivo, os resultados são comparáveis aos dados da literatura, demonstrando a complexidade da doença e da necessidade de novas pesquisas, tendo como objetivo definir o melhor modelo preditor de resistência à IGIV e potencialmente reduzir a principal complicação da DK.
