# Supplementary material for: Doença de Kawasaki: Preditores de Resistência à Imunoglobulina Intravenosa e Complicações Cardíacas: Novas Perspectivas?
Source: Arq Bras Cardiol. 2021 Mar 3;116(3):492–3. [Article in Portuguese] doi: 10.36660/abc.20201353 (PMC8159543; doi:10.36660/abc.20201353)
Supplement: Translation

# Translation: Kawasaki Disease: Predictors of Intravenous Immunoglobulin Resistance and Cardiac Complications: New Perspectives?

**Affiliations:** 1 SalvadorBA Brazil Universidade Federal da Bahia - Faculdade de Medicina da Bahia, Salvador, BA – Brazil

**Keywords:** Mucocutaneous Lymph Node Syndrome/trends, Kawasaki Disease, Coronary Artery/abnormalities, Vasculitis, Children, Immunoglobulins, Intravenous, Drug Resistance

Kawasaki disease (KD) is an acute inflammatory disease of unknown origin and associated with vasculitis. It affects medium-sized vessels. Coronary artery abnormalities (CAA) — aneurysms or dilations — are the main complications of KD, and are currently the most common cause of heart disease acquired in children in developed countries. In the acute phase, the initial standard treatment recommended is intravenous immunoglobulin (IVIG) and aspirin, to reduce the risk of damage to the coronary arteries.^1^ However, 10 to 20% of children with KD do not respond to the initial standard treatment, with recurrent fever within 36 to 48 hours after IVIG infusion. Studies show that the likelihood of KD patients resistant to IVIG to have coronary artery lesions is nine times greater than those sensitive to IVIG. This suggests that there is a critical window to block the inflammatory process and prevent CAA in the long term.^2^

The study by Faim et al.^3^ aimed to identify predictive factors for resistance to intravenous immunoglobulin (IVIG), calculate the effectiveness of Japanese predictive models and characterize the cardiac complications of patients followed up at a single institution.^3^

Of the 48 KD patients included in the study between 2006 and 2018, 17% were diagnosed with atypical KD. All had fever on the day of admission, with a median of five days, and all were treated in the acute phase with IVIG and aspirin, with a median of 6.5 days. Resistance to IVIG has been described in 9 cases (21%), one of which of atypical KD. These findings are consistent with literature data.^4^

For the early identification of patients resistant to IVIG and to enable the early introduction of other treatment strategies, studies have been carried out trying to establish clinical and laboratory criteria that predict IVIG resistance, such as age, albumin dosage, transaminases, hemoglobin, C-reactive protein (CRP), erythrocyte sedimentation rate (ESR), platelets, bilirubins, sodium, and others.^4^ In the study, the authors constructed ROC curves to find predictive factors for resistance and developed a predictive model using multivariate logistic regression. Analyzing the predictive variables of resistance, CRP presented an AUC ROC = 0.789, cut-off point = 15.1 mg/dL, sensitivity (S) = 77.8% and specificity (E) = 78.9%. The ESR presented an AUC ROC = 0.781, a cut-off point = 90.5 mm/h, S = 66.7% and E = 85.7%. The predictive model with the two variables showed p = 0.042 and AUC ROC = 0.790.

Literature data are controversial regarding the use of ESR as a predictor of IVIG resistance. In a meta-analysis, Baeck et al.,^5^ aiming to identify laboratory factors predictive of IVIG resistance, included twelve studies published between 2006 and 2014, analyzing 2,745 patients. Of these, seven studies calculated the ESR effect size as a predictive factor of IVIG-resistant KD. Heterogeneity between these studies was low (Q (6) = 11.001, P>0.001, I2 = 45.459) and the meta-analysis found that the effect size was small for ESR (random effects, 0.150).^5^

In a meta-analysis performed by Xuan Li et al., analyzing 28 studies involving 26,260 patients, about 4,442 patients were diagnosed with IVIG-resistant KD and 21,818 patients were diagnosed with IVIG-sensitive KD. The meta-analysis showed that ESR in the IVIG-resistant group was significantly higher than in the IVIG-sensitive group. However, the strong association between ESR and KD was demonstrated only in two studies in a Chinese population. The same findings were not shown in Koreans, Japanese and non-Asians. The other studies had a weak association between ESR and IVIG resistance.^6^

The guideline on Kawasaki disease published in 2017 by the American Heart Association,^1^ points out that the ESR increases with IVIG and should not be used to assess therapeutic response, and persistently high ESR should not be interpreted as a sign of resistance to IVIG therapy (Class III, LE: C).

Therefore, we must carefully analyze the use of ESR as an independent risk predictor for IVIG resistance in the non-Asian population.

The three models of risk scores developed, namely Kobaysahi et al.,^7^ Sano et al.^8^ and Egami et al.,^9^ validated for the Japanese population,^7^^–^^9^ do not work properly in western, ethnically mixed and Chinese populations.^10^^–^^14^ A similar situation was observed by Faim et al.,^3^ where the respective models validated in Japan did not perform well either.^3^

It is worth noting that in the study by Faim et al. about 25% of the patients had coronary involvement, 40% of them had aneurysm formation, in addition to other cardiovascular complications. CAA and other cardiovascular complications in the acute phase, including myocarditis, cardiogenic shock and pericardial effusion, are described in the literature.^1^^,^^2^^,^^4^

Regardless of the size of the sample analyzed and its retrospective nature, the study findings are comparable to literature data, demonstrating the complexity of the disease and the need for further research aiming to define the best predictive model of IVIG resistance and potentially reduce the main KD complication.
